# 2011 Psychological mechanisms linking food insecurity and obesity

**DOI:** 10.1017/cts.2018.259

**Published:** 2018-11-21

**Authors:** Candice A. Myers, Stephanie T. Broyles, Corby K. Martin, Peter T. Katzmarzyk

**Affiliations:** Pennington Biomedical Research Center – LA CaTS

## Abstract

OBJECTIVES/SPECIFIC AIMS: The current pilot study will use a mixed methods approach to investigate the role of psychological mechanisms in the relationship between food insecurity and obesity. We will be the first to assess 4 key psychological constructs (delay discounting, grit, future time perspective, and subjective social status) in a sample of food secure and food insecure adults with and without obesity. The specific aims are: (1) Examine associations among psychological mechanisms, food security status, and body mass index (BMI); and (2) Collect qualitative data on psychological mechanisms linking food insecurity and BMI. METHODS/STUDY POPULATION: This is a cross-sectional, observational pilot study that will be conducted in the local Baton Rouge community. The target study sample is 56 food secure and food insecure women and men aged 18–49 years with a BMI of 20.0 kg/m^2^ or greater. Independent (grouping) variables are food security status and BMI. Primary endpoints are 4 psychological constructs measured via questionnaires: (1) delay discounting, (2) grit, (3) future time perspective, and (4) subjective social status. We will also assess a number of key covariates, including health literacy, sociodemographics, food assistance use, and dietary quality. Semistructured, in-depth interviews will be conducted in a subsample of 12 participants. RESULTS/ANTICIPATED RESULTS: For quantitative data, we will test for significant associations between food insecurity, obesity, and selected psychological mechanisms via bivariate correlations and linear and logistic regression models. Qualitative data will be analyzed to identify key themes and concepts that conceptually link the aforementioned psychological mechanisms to food insecurity and obesity. Analyzed qualitative data will be triangulated with quantitative findings. DISCUSSION/SIGNIFICANCE OF IMPACT: This pilot study will examine the role of psychological mechanisms in the relationship between food insecurity and obesity. Moreover, we are gathering data to identify potentially new intervention targets that will be used to develop intervention strategies aimed at reducing health disparities by effectively promoting weight management among low socioeconomic populations.

